# The Relationship between Executive Functions and Metacognition in College Students

**DOI:** 10.3390/jintelligence11120220

**Published:** 2023-11-30

**Authors:** Mengjiao Wu, Christopher A. Was

**Affiliations:** 1 College of Foreign Language, Shanghai Maritime University, Shanghai 201306, China; mjwu@shmtu.edu.cn; 2 The Psychological Sciences, Kent State University, Kent, OH 44240, USA

**Keywords:** executive functions, metacognition, updating

## Abstract

This study investigated the relationship between executive functions and metacognition. Both constructs have been well-studied, but little research has focused on their connections. The goal of the current investigation was to increase the understanding of the relationship between metacognition and executive functions by assessing the relationships between metacognitive monitoring accuracy and the three component executive functions (updating, inhibition, and shifting) among college students. Metacognitive monitoring accuracy was measured using a knowledge monitoring accuracy (KMA) test. The three components of executive functions, updating, inhibition, and shifting were measured, respectively, using the ABCD updating task, the Stroop color–word interference test, and the letter–number task. The Tower of Hanoi task was used to measure the complex executive functions (inhibition and updating). Correlation and regression analyses were performed to examine the relationships. The results indicate that updating is the only component executive function that significantly correlated with metacognitive monitoring, suggesting that metacognition—specifically, metacognitive monitoring—is associated with at least one component of executive functioning.

## 1. Introduction

Existing literature shows that students require specific learning skills and strategies for successful self-regulated learning (see Bjork et al. 2013, for a review). Two constructs in psychological literature—executive functions and metacognition—are often described as the foundation of self-regulated learning. These two uniquely developed constructs explain different phenomena and are applied in different practices. However, there are some similarities that overlap between the two. Unfortunately, little attention has been paid to the individual differences in and the association between executive function and metacognition, despite the possible overlap. Put differently, if executive functioning and metacognition are associated, then individual differences in one should be related to or predict individual differences in the other. This study explored the associations between metacognition and the three fundamental component executive functions to promote a more specific and comprehensive understanding of these two constructs. In the following, we provide a summary of the constructs and our theoretical reasoning for the relationship between the two. We begin with executive functions.

The concept of executive functions was first proposed in Baddeley and Hitch’s (1974) influential multi-component model of working memory, which proved to be effective in explaining a range of cognitive phenomena. The original model comprised three components, including central executive, phonological loop, and visuospatial sketchpad, among which, the central executive is responsible for the control and regulation of cognitive processes. Complex functions of the central executive were identified, such as the temporary activation of long-term memory, coordination of multiple tasks, planning, shifting between tasks, maintaining a memory for a short term, and selecting to attend to or inhibit a specific task (Baddeley 1998).

The complex functions were categorized by Shallice (1988) as executive functions: the management of a variety of self-regulatory cognitive processes to achieve a cognitive goal, which include planning, solving problems, dealing with unknown situations, resisting interference, directing and maintaining attention, and more. Complex executive functions are considered higher-order functions that are composed of at least two component executive functions. The component executive functions are distinguished from the complex executive functions as the minimum and fundamental core unit of the executive functions that work together to support complex executive tasks.

Miyake et al. (2000) identified three component executive functions: shifting, updating, and inhibition. Shifting, or task-switching, refers to shifting back and forth between tasks, operations, and mental sets (Monsell 1996). The number–letter task (Rogers and Monsell 1995) and the color-shape task (Miyake et al. 2004) are believed to be effective in assessing shifting. Updating involves monitoring, coding incoming information, and replacing old information with new information (Morris and Jones 1990). The essence of the updating function lies in information manipulation instead of simple information storage. It is usually measured by dual-task performance, for instance, the operation span task (Miyake et al. 2000). Inhibition involves deliberately suppressing irrelevant information from entering working memory. The Stroop task (Stroop 1935) is typically used to measure this ability. Although the component functions as described appear to be unique and diverse, the question of unity and diversity of the components (Miyake et al. 2000) continues to be a point of debate.

This debate has attracted much attention (e.g., Hughes 1998; Welsh et al. 1991; Senn et al. 2004). The study conducted by Miyake et al. (2000) made a strong claim on both the unity and diversity of executive functions. All together, nine tasks were completed with each component executive functions (updating, shifting, and inhibition) measured by three tasks. The results from the confirmatory factor analysis suggested that the three component executive functions share some underlying commonality but are also distinguishable. The findings from a series of structural equation modeling (SEM) analyses indicated that the three component executive functions uniquely contribute to the performance of complex executive tasks. For instance, the latent construct of updating was found to be most predictive of the operation span task, and the Wisconsin Card Sorting Test (WCST) had the greatest factor loading on the latent factor of shifting ability. Therefore, we believe research on the relationships between the three component executive functions, updating, shifting, and inhibition, and metacognition could provide insight into the relationships between more complex executive functions and metacognition, and help better understand those relationships. This is the reason why the current study examined the relationship between executive functions and metacognition through component executive functions.

Necessary for academic achievement, Shallice (1988) proposed that executive functions make up a variety of self-regulatory cognitive processes to achieve cognitive goals, which include planning, solving problems, dealing with unknown situations, resisting interference, directing and maintaining attention, and more. Plenty of evidence suggests a positive relationship between higher-order (complex) executive functions and school achievement. For example, McClelland et al.’s (2007) study indicated that preschoolers with better executive functions (e.g., planning) outperform the ones with lower executive functions in the emergent literacy, vocabulary, and mathematics achievement, and even the growth in those skills over the prekindergarten year. Roebers et al. (2012) found individual differences in executive functioning significantly related to academic outcomes, literacy and mathematics in specific among young elementary school children. A few other investigations reported similar results. Students with higher executive function abilities were proved to have higher math achievements (Bull and Scerif 2001). Indeed, executive functions are often touted as predictors of academic achievement (Best et al. 2011; Clair-Thompson and Gathercole 2006; Lan et al. 2011). However, more specific patterns have emerged with closer scrutiny of the data and examination of the component executive functions. For instance, updating showed a stronger association with educational achievement than the other two component functions, especially in reading/writing abilities (Clair-Thompson and Gathercole 2006), while math achievement was found to be best predicted by inhibition among the three component executive functions (Espy et al. 2004).

The review of executive functions, both component and complex, indicates that executive functions are clearly related to academic achievement and complex cognitive processing. The second construct of interest in our investigation is metacognition. There is a large corpus of literature regarding metacognition and the relationship between metacognition and academic achievement (see Hacker et al. 1998). Next, we describe the construct of metacognition, and explain our focus on metacognitive knowledge monitoring.

Metacognition refers to the knowledge of and control over one’s own cognition (Flavell 1979) and is often perceived as a higher-order self-reflective cognitive process that monitors and regulates cognition (Schneider 2011). Garcia and Pintrich (1994) regarded metacognition as a set of common skills that is significantly associated with academic achievement. Three facets of metacognition have been identified and studied: metacognitive knowledge, metacognitive monitoring, and metacognitive control (Dunlosky and Metcalfe 2008). Metacognitive knowledge refers to people’s declarative knowledge about cognition; metacognitive monitoring is the evaluation of an ongoing cognitive process; and metacognitive control refers to the control and regulation of the cognitive activity usually based on the result of the monitoring. Multiple frameworks have been developed to portray how metacognition works, and how it impacts cognition. Among them, Nelson and Narens’ (1990) two-central-dimension framework is influential and widely accepted. It includes two interactive levels: the meta-level and the objective level (the ongoing cognitive process). In this model, the flow of information runs from the object level to meta-level when monitoring, and then runs from the meta-level back to object level so that the controlling can take effect if modification is needed according to the monitoring results. Their framework suggests that people are capable of monitoring and managing thinking with higher-level representation (Dunlosky and Metcalfe 2008). The framework also indicates that effective monitoring is the prerequisite of effective control. Without accurate monitoring, it will be difficult to make proper judgments and effective control of the on-going cognitive processes (Dunlosky and Metcalfe 2008). For instance, if a learner underestimates how much he/she has learned, he/she may spend unnecessary time and effort on the learning materials that are already well learned. If a learner overestimates how much he/she has learned, he/she may end the learning process before the target learning materials are well learned and end up with lower learning outcomes. Accurate monitoring is the necessary antecedent of appropriate and efficient control actions (Nelson and Narens 1990; Renner and Renner 2001). The current investigation focused on metacognitive monitoring when assessing the relationship between metacognition and executive functions.

Metacognition was also found to be highly related to learning performance, including more specific learning outcomes and general academic achievement, such as reading (Van Kraayenoord and Schneider 1999), chemistry (Rickey and Stacy 2000), science (Topçu and Yılmaz-Tüzün 2009), and learning abilities (Ruban 2000; Trainin and Swanson 2005). Metacognitive knowledge was found to increase from less to more successful students (Peklaj and Pecjak 2002), and higher achievers presented more awareness of metacognitive knowledge and control than lower achievers (Van Kraayenoord and Schneider 1999). Empirical research has repeatedly shown that the accuracy of metacognitive monitoring is highly associated with memory and learning performance across age and tasks (Zimmerman and Kitsantas 1999; Maki 1998; Dunlosky et al. 2005; Roderer and Roebers 2010). All this evidence indicated metacognition’s influence on general academic achievement.

Metacognitive monitoring accuracy is commonly measured by the judgments of learning, feeling of knowing, and judgments of confidence. In studies using these types of judgments, participants are asked to make subjective and explicit evaluation of their learning, knowledge, or confidence. Monitoring judgments are believed to be made based on multiple criteria, including cue familiarity, target accessibility, retrievability, and task difficulty (Koriat and Levy-Sadot 2001; Dunlosky and Metcalfe 2008). The judgments are also likely to be affected by various processes, such as searching in long-term memory, storage of the retrieved information, updating the content of working memory, and so on (Koriat and Levy-Sadot 2001). All of them contribute to the complex nature of metacognitive monitoring that it is multiply determined and influenced by different mechanisms (Koriat and Levy-Sadot 2001; Nelson et al. 1984).

We now turn to the similarities in the constructs of executive functions and metacognition that provided the impetus for the current study.

A sparse but growing body of research has focused on the relationships between executive functions and metacognition from both theoretical and empirical perspectives, and a strong correlation is suggested. One important practical similarity between executive functions and metacognitive knowledge monitoring is that both are found to be effective predictors of academic achievement among children and adults (e.g., Best et al. 2011; Clair-Thompson and Gathercole 2006; Lan et al. 2011; Ruban 2000). Both constructs are closely related to self-regulation, and what is more, both showed similar developmental patterns of continuous improvements from early childhood to adolescence (Roebers 2017; Hughes and Ensor 2011; O’Leary and Sloutsky 2019).

Theoretically, the similarities between them are evident when comparing two influential theories in cognitive psychology (Fernandez-Duque et al. 2000). In Nelson and Narens’s (1994) two-level metacognitive model, the meta-level monitors the object level by receiving bottom–up information, and controls it through offering a top–down output. A similar information flow is observed in Norman and Shallice’s (1986) theory of executive functions, in which the executive system regulates (activates or inhibits) the lower-level information (schemas) with intentions (top–down) after receiving feedback from schemas. According to the review of Fernandez-Duque et al.’s (2000), executive functions and metacognition are closely related. Both executive functions and metacognition receive information from the object level and enable the top–down regulation of cognitive processes. Both can be used to explain the process of voluntary actions. They both contribute to the flexibility and independence of cognitive processes by making the processes less vulnerable to external stimuli, which are necessary for problem solving, decision making, strategy application, and may result in automatic responses.

The integrated model proposed by Demetriou and colleagues (Demetriou and Kazi 2001; Demetriou et al. 2002) regarding the relationship between cognitive and metacognitive processes also suggests the relationship between executive functions and metacognition. According to their model, there are three fundamental levels of mind organization: a basic processing potential related to the processing system (such as inhibition and working memory), an environment-oriented level which involves cognitive systems representing and processing information from the environment, and the metacognitive system. The three levels are believed to be distinct and dynamically intertwined, which theoretically suggests the interrelations between the executive functions and metacognition.

Fernandez-Duque et al. (2000) also suggested that executive functions and metacognitive skills share certain functional locations in the brain. The neural circuits underlying executive attention have been identified in the frontal areas, including the anterior cingulate and supplementary motor area, the dorsolateral prefrontal cortex, the orbitofrontal cortex, etc. (Bush et al. 2000; Posner and McCandliss 1999; Moriguchi and Hiraki 2013). A few neuro-imaging studies also found metacognition associated with the frontal cortex (Shimamura 1995; Kikyo et al. 2002; Souchay and Isingrini 2004; Pannu and Kaszniak 2005; Fleming and Dolan 2012), suggesting that executive functions and metacognition share the same functional location in the brain. Fernandez-Duque et al. (2000) believed the findings and techniques in cognitive neuroscience would help promote research in metacognition. We expect that relating behavioral patterns associated with executive functions and metacognition will benefit the understanding of both constructs.

Despite so many similarities displayed between executive functions and metacognition, little research has been conducted to examine the possible relationship between the two constructs (Roebers 2017). One contributing factor to this lack of empirical investigation of this relationship is that they are investigated in different research channels with different foci (Fernandez-Duque et al. 2000). According to Fernandez-Duque et al. (2000), metacognition has been mostly studied in regards to metacognitive knowledge, with a concentration on metacognitive development in childhood period; and studies in executive functions, largely advanced by cognitive psychologist and cognitive neuroscientists, have often focused on normal adults and patients with brain impairment, and explored the link between cognitive control processes and brain structures.

In order to bring more attention to the connection between executive functions and metacognition, Roebers (2017) proposed a unifying framework of cognitive self-regulation that integrates these two constructs, and argued that they share more common grounds than differences. Among the other studies that have investigated this relationship, some worked with adults (Bekci and Karakas 2006; Perrotin et al. 2007; Perrotin et al. 2008) and found evidence to support the association between executive functions and metacognitive monitoring. Specifically, the study conducted by Perrotin et al. (2008) investigated the relationships between metacognitive monitoring—episodic feeling of knowing—and complex and component executive functions among healthy older adults (mean age: 70.33 years). The results of the correlations and regression analysis showed that the feeling of knowing accuracy was more reliant on executive functions than memory performance, fluid intelligence, or processing speed. Among the three component executive functions, shifting was observed as the main and only predictor of feeling of knowing accuracy. Another study (Souchay et al. 2000) compared the feeling of knowing accuracy and executive functions among healthy older (mean age: 72.04 years) and younger (mean age: 24.25 years) adults. The feeling of knowing accuracy was found to decrease with the age-related decline of executive functioning. This suggested a correlation between the feeling of knowing accuracy and executive function.

Some recent investigations focused on this relationship in children’s development. Kälin and Roebers (2022) investigated the association between components of executive functions and metacognitive monitoring among 5- to 8-year-olds, and found that inhibition was the only component that significantly related to later metacognitive monitoring. However, another study investigating the same relationship yielded no significant association between component executive functions and metacognitive monitoring among 4- to 6-year-old children, but inhibition was found to be significantly related to the latencies of monitoring judgments (Kälin and Roebers 2020). It seems that the link between inhibition and monitoring only exists in younger children before formal schooling. Souchay and Isingrini (2004) observed the age differences in metamemory control performance, and a partial correlation between metamemory control and executive functions. Roebers et al. (2012) examined associations between executive functions and metacognition among young elementary school children, both cross-sectionally and longitudinally, and reported that executive functions significantly and directly related to metacognitive control but not monitoring, and executive functions could be used to predict later metacognitive control. Another study (Bryce et al. 2014) found that the association was higher in 5-year-old children than 7-year-olds, suggesting that executive functions and metacognition are not identical skills, but executive functions could be “necessary but not sufficient” for metacognition. However, there are also studies showing no significant link between executive functions and metacognition among children (Destan and Roebers 2015; Marulis and Nelson 2021).

In further discussing the relationship between executive functions and metacognition, some researchers argued for a directional relation. For example, the flexible shifting between meta-level and object-level cognitive processes is argued to be necessary for monitoring and control (Dunlosky and Bjork 2008). Kuhn and Pease (2010) argued for the important role of inhibition as the use of previously used strategies needs to be inhibited in order to allow for new learning strategies when an individual develops flexible strategy use. Kälin and Roebers (2020) worked on exploring a possible bidirectional relation between inhibition and monitoring among children from kindergarten to second grade, and the results suggested a critical role of inhibition in early childhood for later monitoring skills. Thus, Kälin and Roebers (2022) came to an assumption that inhibition serves as a vital antecedent for later monitoring in early childhood.

Most research on the relationship between executive functions and metacognition among adults was limited to the use of complex executive functions tasks, and focused on the same aspect of metacognitive monitoring, i.e., the feeling of knowing. When a different aspect of metacognitive monitoring was measured, no association with executive functions was found (e.g., Pennequin et al. 2010). Component executive functions were more frequently used in investigating this relationship among children. However, the results are not consistent (Souchay and Isingrini 2004; Bryce et al. 2014).

Thus, regardless of these efforts, the relationship between executive functions and metacognition remains unclear. The current study attempted to contribute to the literature by exploring the correlations between metacognitive monitoring, component executive functions (updating, shifting, and inhibition), and one complex executive function among young adults for a more comprehensive, developed, and stable relationship between metacognition and executive functions.

Specifically, we hypothesized the relationships among metacognitive monitoring and the component executive functions of inhibition, shifting, and updating. Based on the models of metacognition and executive functions described previously, we hypothesized that inhibition is a process needed during metacognitive processing to inhibit irrelevant thoughts and information so that one can focus on the target cognitive process. Shifting was hypothesized to be critical in the ability to switch back and forth between the object-level and meta-level processing, which guarantees the processing of both tasks. Updating was hypothesized to be associated with metacognitive monitoring as monitoring requires working memory to preserve task-relevant information and metacognitive control requires updating of the object level.

Due to the complex nature of metacognitive monitoring—as it is affected by multiple processes and different criteria (Koriat 1993; Koriat and Levy-Sadot 2001; Dunlosky and Metcalfe 2008)—complex executive functions might be associated with metacognitive monitoring accuracy by allowing for flexible switches between various underlying mechanisms and operations, updating of information in the system, and inhibition of irrelevant information (Perrotin et al. 2008).

## 2. Materials and Methods

### 2.1. Participants

Seventy-three undergraduate college students enrolled in an educational psychology course at a large Midwestern university participated in the study for a course credit. Due to computer errors, 12 participants had missing data from the Tower of Hanoi task. All participants were sophomores or juniors, with a mean age = 20.12 years.

### 2.2. Materials

#### 2.2.1. Knowledge Monitoring Accuracy

Metacognitive knowledge monitoring was measured using a knowledge monitoring accuracy (KMA) test (e.g., Isaacson and Was 2010; Tobias and Everson 2000), designed to measure one’s ability to accurately judge their knowledge of word meanings. The KMA began with the random presentation of 50 English words (see Data Availability Statement) on the computer screen sequentially. When each word was presented, participants were instructed to make a first-order judgment of knowing (JOK) for each word by rating if they know the meaning of the word from 0 (not known) to 100 (known). After rating the judgments of knowing for all 50 words, participants’ knowledge of those words was tested in a multiple-choice vocabulary test with five possible synonyms, from which they chose the word they believed to have the same meaning as the target word. Participants then made a second-order judgment of confidence (JOC) immediately after each word test response, in which participants indicated their confidence in the accuracy of their response on a scale of 0 (from no confidence) to 100 (complete confidence).

#### 2.2.2. Updating

The ABCD task (Kyllonen and Christal 1990; Was 2007; Was and Woltz 2007; Woltz 1988) was used to measure updating. This task was composed of 18 trials, each consisting of three statements presented sequentially on the computer screen. First, the statement presented the order of letter “A” in relation to “B” in Set 1 (e.g., “A is followed by B”). The second presented the order of letter “C” in relation to “D” in Set 2 (e.g., “C is preceded by D”). The last one stated the order of Set 1 in relation to Set 2 (e.g., “Set 1 precedes Set 2”). The order of the three statements and the expression of the order in each statement were random. The processing time for each statement was self-paced but limited to 20 s. After the third statement, participants were presented with a response screen and required to select a response (e.g., BADC) from eight alternatives as quickly as possible. The accuracy and reaction time feedback were presented after each response. Experimental trials began after 3 practice trials.

#### 2.2.3. Inhibition

A computerized version of the Stroop color–word task, adopted from the Stroop color–word interference test (Stroop 1935), was used as a measure of inhibition (Archibald and Kerns 1999). The task is designed to measure the ability to inhibit prepotent response tendencies that are incongruent with the task goal. During the task participants were visually presented the names of colors displayed in colors (e.g., the word “red” presented in green font). On each trial, participants were to respond to the font color by pressing a corresponding-colored key on the computer keyboard. Seventy-five percent of the trials were congruent trials that presented the color name in the font of the same color (e.g., the word “red” presented in red font). The other 25% of the trials were incongruent trials that presented the color name in another font (e.g., the word “red” presented in green font). Congruent and incongruent trials were mixed and presented in a random order. The accuracy of these trials required the inhibition of the stimulus-bound response—to respond to the font color of the word rather than the written color name. On each of the 128 sequentially presented trials, participants were instructed to respond by clicking the keys indicated by the colored stickers (red, green, yellow, and blue) on the keyboard. Participants were instructed to respond as quickly as possible while trying to avoid errors. The reaction time and accuracy feedback were displayed after each trial. Twenty practice trials were provided for warm-up, but the results were excluded from the data. The dependent measure was the RT difference between the correctly answered congruent and incongruent trials.

#### 2.2.4. Shifting

The number–letter task, adapted from Rogers and Monsell (1995), was used to measure shifting. The task was composed of consistent trials, in which the rule remained the same, and inconsistent trials, in which the rules changed from trial to trial. Participants were presented with a number–letter combination (e.g., 2M), one at a time, in one of four quadrants on the computer screen. If the combination was presented on the top row (top left or top right), participants were instructed to respond whether the number was odd or even by pressing the corresponding key on the keyboard; if the combination was presented on the bottom row (bottom left or bottom right), participants were required to respond whether the letter was a consonant or a vowel. The number–letter combination was presented only on the top row for the first block of 32 trials, only on the bottom row for the second block of 32 trials, and in a random manner in the four quadrants for another 128 trials. The first and second blocks of trials required no mental shift of task rules, but it was required in the last 128 trials when the position of the combination was randomized. Using the last 128 trials, shift cost was measured by subtracting the mean reaction time inconsistent trials (a rule change occurs) and consistent trials (no rule change occurred).

#### 2.2.5. Complex Executive Functions

A computerized version of the Tower of Hanoi was used to measure the complex executive functions (Goldman-Rakic 1987; Goel and Grafman 1995; Roberts and Pennington 1996). In the task, participants were presented with a target configuration of four disks of varying size on three pegs horizontally placed on the top 1/3 of the computer screen, and a different starting configuration of four disks on three pegs horizontally placed in the bottom of the computer screen. Participants were instructed to make the starting configuration look like the upper target configuration by moving the disks in the starting configuration with a computer mouse. A set of rules was imposed on this task: only a certain number of moves were allowed for each trial as instructed before the trial started; only one disk could be moved at a time, and a larger disk could not be put on top of a smaller disk. If the participants were not able to solve the puzzle within the allowed number of moves, a warning would appear informing that they had not completed the task in the allotted number of moves, and the same trial would restart until it was successfully completed. There were all together six trials with the first trial set as a practice trial. The dependent measure was the total number of moves taken to complete the last five trials, including the wrong moves.

### 2.3. Procedure

Participants completed the five tasks in two 1 h sessions. KMA and the Stroop color–word task were administered on day one, and the ABCD task, letter–number task, and Tower of Hanoi were completed on the second day.

## 3. Results

### 3.1. Knowledge Monitoring Accuracy

The mean accuracy of the multiple-choice portion of the KMA (the number of words participants correctly identified) was M = 51.50 with a SD = 10.78 (Cronbach’s α = 0.68). On average, participants provided a JOK of 52.25 (SD = 13.37) and a JOC of 50.52 (SD = 13.54) for the 50 words. To determine individual differences in first-order judgement accuracy and second-order judgment accuracy, we first calculated correlations between multiple-choice accuracy (proportion correct), and the first- and second-order judgments for each individual participant. Higher correlations indicate more accurate JOKs and JOCs, respectively. The mean correlation between multiple-choice accuracy and first-order JOKs was M_r_ = 0.33, SD_r_ = 0.15, and for second-order JOCs, M_r_ = 0.46, SD_r_ = 0.20.

In the literature on metacognition, it is common to present and analyze data using signal detection theory (SDT) metrics. In SDT, data are often dichotomous. To align with the convention and present data in such a manner, we transformed the rating to dichotomous variables: when the rating provided was higher than 50, it was interpreted as knowing or being confident in the performance, whereas if the rating was lower than or equal to 50, it was interpreted as being not knowing or being unconfident in the response. For each word in the KMA, the result of the monitoring accuracy of the JOK and JOC could fall into one of the four possible outcomes: a hit, correct rejection, miss, or false alarm. Using terminology common to the signal detection theory, Table 1 presents descriptive statistics for JOKs and JOCs. Regarding (1) hits: participants responded that they knew the meaning and got the correct answer on the vocabulary test; (2) false alarms: participants responded that they knew the meaning but made the incorrect choice on the test; (3) misses: participants responded that they did not know the meaning but chose the correct answer on the test; (4) correct rejections: participants responded that they did not know the answer and gave an incorrect answer on the test. Both hits and correct rejections indicate accurate metacognitive knowledge monitoring, and the other two indicate inaccurate knowledge monitoring.

The frequencies of each outcome were used to indicate the accuracy of knowledge monitoring: higher frequencies of hits and correct rejections were regarded as higher accuracy, whereas higher frequencies of misses and false alarms indicated a lower accuracy. Therefore, the frequency of accurate monitoring was calculated by adding the frequencies of hits and correct rejections, and the frequency of inaccurate monitoring was represented by the total frequency of misses and false alarms. The means and standard deviations of the above frequencies are presented in Table 1. In addition to the frequencies, The Goodman–Kruskal gamma correlation (Goodman and Kruskal 1954) was also calculated and used to represent the accuracy of metacognitive judgments, as it is recommended as a reliable measure of relative accuracy of metacognitive judgments (Nelson 1984; Schraw and Moshman 1995; Isaacson and Was 2010). A JOK gamma coefficient and a JOC gamma coefficient were calculated for each participant by computing the correlation between one’s judgments about items and performance on the same items in a test (Goodman and Kruskal 1954):

(1) $$\gamma =\frac{ad-bc}{ad+bc}$$

Note: *a* = frequency of hits, *d* = frequency of correct rejections, *b* = frequency of false alarms, *c* = frequency of misses.

The gamma coefficient ranges from −1.0 to +1.0. A positive gamma score represents accurate monitoring, while a negative gamma score indicates inaccurate monitoring. Table 1 presents the descriptive data of the frequencies of the four outcomes, and the gamma scores in JOK and JOC. Therefore, in this study, the accuracy of metacognition was indicated by both the frequencies of accurate and inaccurate judgments, and the gamma coefficients in the judgments of knowing (JOK) and judgments of confidence (JOC).

### 3.2. Cognitive Task Data Analysis

Individual differences in executive functions were measured using the four cognitive tasks described above, which, respectively, tap the abilities of updating, inhibition, shifting, and the complex executive functions. The component EF skill of updating was measured with the ABCD task. Each participant received two scores: the test accuracy (M = 0.81, SD = 0.14, Cronbach’s α = 0.68) and reaction time (M = 2815.32 ms, SD = 1175.06 ms, Cronbach’s α = 0.87).

Inhibition was measured with the Stroop color–word task. Four outcome variables were captured from the task: accuracy and reaction time (RT) in both congruent and incongruent conditions. Among them, two were theoretically expected to tap the inhibition ability: accuracy and reaction time in the incongruent conditions, as inhibition is needed to suppress the dominant response of the color word to accurately respond with the font color in the task. To accurately measure inhibition, it is typical to only use correctly answered trials for RT analysis (Miyake et al. 2000). The descriptive statistics of the four variables presented in Table 2 showed that participants had performed well on the congruent trials (mean accuracy = 0.98, SD = 0.19), but their performance dropped dramatically on the incongruent trials (mean accuracy = 0.06, SD = 0.14). Indeed, 30 of the 73 participants did not respond correctly to any of the incongruent trials. Therefore, we chose to use the difference between reaction time for all congruent trials and incongruent trials as our outcome variable for the Stroop task.

Participants’ mean reaction times in both consistent and inconsistent trials in the number–letter task are presented in Table 3. The mean reaction time in the inconsistent trials was expected to be longer than in the consistent trials. The difference between the mean reaction time in consistent conditions and inconsistent conditions was calculated to represent the cost of shifting between different rules. The greater the difference was, the more the participant was influenced by the rule changing, the less competitive the participant was in the ability of shifting. The mean reaction time in the consistent condition was 798.73 ms (SD = 237.76 ms), and in the inconsistent condition, it was 1139.99 ms (SD = 274.95 ms). The mean difference of reaction between consistent and inconsistent conditions was 409.26 ms (SD = 321.00 ms).

Complex executive functioning was measured with the task of the Tower of Hanoi, which is believed to tap both inhibition and updating skills (Miyake et al. 2000). The total number of moves to complete the five experimental trials, including failed attempts and wrong moves, was summed. The mean of the total moves to complete the five trials was 50.85 (SD = 7.81).

### 3.3. Results of Correlations between Metacognition and Executive Functions

Table 4 presents the correlations between the accuracy of the KMA and performance on the four cognitive tasks. Individual word accuracy/JOK and word accuracy/JOC correlations were significantly correlated with ABCD reaction time, with r = −0.25, *p* = 0.035 and r = −0.29, *p* = 0.013, respectively. The JOK gamma significantly correlated with ABCD accuracy: r = 0.24, *p* = 0.045 and ABCD reaction time, r = 0.33, *p* = 0.004. The analyses revealed no other significant correlations between the KMA and performance (accuracy and reaction time) on the other measures of executive function.

We also conducted two regression analyses. In the first, all executive function measures were predictors of the criterion word accuracy/JOK correlations, and in the second, the criterion was word accuracy/JOC correlations. Table 5 presents the regression coefficients and correlation outcomes of both regressions. In the first regression, no predictors accounted for significant variance in the criterion and the model was not significant, R^2^ = 0.09, F (5,53) = 1.09, *p* = 0.377. In the second model, with word accuracy/JOC correlations as the criterion (R^2^ = 0.20, F (5,53) = 2.71, *p* = 0.030), only ABCD accuracy (b = −0.36. t(58) = −2.18, *p* = 0.034), and ABCD reaction time (b = −0.53, t(58) = −3.26, *p* = 0.002) accounted for significant variance.

We conducted similar regression analyses with gamma for JOKs and JOCs as with the dependent variables. The regression with gamma for JOKs as the dependent variable was not significant (F < 1), nor was the regression model with gamma for JOCs as the dependent variable (F < 1).

Overall, metacognitive monitoring accuracy is significantly associated with the performance on an updating task, suggesting a significant and positive correlation between metacognitive monitoring and the executive function of working memory updating. Confidence judgements were more strongly related to updating than the judgments of knowing. The results also indicated that there was no association between metacognitive monitoring and the component executive functions of inhibition, shifting, and complex executive functions practiced in the task of the Tower of Hanoi.

## 4. Discussion

In order to gain a better understanding of the relationship between metacognitive monitoring and executive functions, the current study examined the correlations between metacognitive monitoring, three component executive functions—updating, inhibition, and shifting—and a complex executive function task. Metacognitive knowledge monitoring was measured using a knowledge monitoring assessment. The three component executive functions were measured employing three unique tasks designed to capture specific components of executive functions. Because of the similarities between metacognition and executive functions suggested by the extant literature, we hypothesized that metacognition is associated with the component executive functions: updating, inhibition, and shifting. However, the current results did not support our hypothesis. Instead, correlation analyses revealed significant correlations between metacognitive knowledge monitoring and updating, but no significant correlation between metacognitive knowledge monitoring and the other two component executive functions or the complex EF measured by the Tower of Hanoi. Confidence judgments—JOCs—were more strongly related to updating than JOKs. Put differently, the results indicated that among college students, metacognitive monitoring highly correlates with only one of the component executive functions—updating.

One explanation for the current findings is that metacognitive monitoring requires constant updating and maintenance of the progress of on-going object-level processing. According to Nelson and Narens’ (1990) model of metacognition, in order to efficiently control object-level cognitive processing, the meta-level (metacognition) must continuously monitor and update the performance of the on-going object level for evaluation and necessary manipulation. Meanwhile, since monitoring is usually performed simultaneously with the object-level processing, a strong updating skill is needed to maintain and update the goals of both processes. We suspect that JOCs were more strongly related to updating than JOKs because participant JOCs were more highly correlated with accuracy on the multiple-choice word identification component of the KMA. During the initial JOK, participants must report a judgment of their feeling of knowing the word in the absence of context. Then, after the multiple-choice test they should have updated their judgment of knowing the word based on the ability to identify the correct synonym. Therefore, it is possible that participants are updating both their judgments about their knowledge and whether they chose the correct synonym. The results indicate the primary importance of updating skills in accurate metacognitive monitoring. Demetriou et al.’s (2002) multi-level model can also be used to explain the important role of updating in metacognition, as working memory is identified as an important predictor of thinking and problem-solving abilities in that model, and problem-solving skills are associated with metacognition (Pennequin et al. 2010).

Contrary to our hypothesis, our study found no correlation between metacognitive monitoring and the other two components of executive functions (shifting and inhibition). This suggests that the skills of shifting and inhibition are not strongly involved in performing accurate metacognitive monitoring. Although we expected shifting to be related to metacognitive monitoring, it is understandable that no correlation was found. One might expect that attending to the object level and then attending to the meta-level might represent a type of task switching. However, our results suggest that the process of updating is more foundational to knowledge monitoring than the ability to efficiently switch tasks.

The insignificant correlation between inhibition and metacognitive knowledge monitoring is also easily understood. It is not likely that the ability to accurately judge one’s knowledge at a given moment requires the ability to inhibit a prepotent response. We can envision that if an individual has a bias toward a particular response (e.g., a pessimistic tendency to underestimate one’s knowledge), accurate knowledge monitoring would require the ability to inhibit a response of “don’t know” when asked the meaning of a word. However, our results do not support this assumption.

The Tower of Hanoi puzzle is suggested to tap the component executive functions of inhibition (Roberts and Pennington 1996) and updating (Goldman-Rakic 1987); however, in the current study, we did not find a significant correlation between metacognitive knowledge monitoring and performance on the Tower of Hanoi. Although these results seem to be inconsistent, they are supported by the literature. Miyake et al.’s (2000) findings shed light on this “inconsistency”. The Tower of Hanoi is believed to tap more than the component executive functions. It also captures planning ability (Arnett et al. 1997) as it involves mentally mapping out a series of moves before taking actions. To execute the careful planning necessary for the successful completion of this task, strategy use is necessary (Simon 1975). Two strategies are found to be the most frequently used and studied: the goal recursion strategy and the perceptual strategy. The goal recursion strategy was believed to be the closest to the concept of “planning” as it involves goal management and maintaining a set of sub-goals in working memory. Thus, updating is believed to be involved in applying the goal recursion strategy. The perceptual strategy involves simply making the next move that will help approach the goal. To fulfill the sub-goals, actions will need to be taken even if they seem to be temporarily distancing one from the goal. Inhibition is thus assumed to play an important role in using the perceptual strategy to solve conflicts between the goal and sub-goals in the Tower of Hanoi. Therefore, the Tower of Hanoi might not be perceived as a “planning” task (Goel and Grafman 1995). Miyake et al. (2000) suggested that if the goal recursion strategy is used in solving the Tower of Hanoi, updating skills is likely to be more dominantly involved, whereas if the perceptual strategy is applied, inhibition skills might be more practiced. However, since the goal recursion strategy is more demanding, it is usually not preferred. The perceptual strategy, which requires more inhibition, is more likely to be used spontaneously (Goel and Grafman 1995) in TOH. Therefore, the results we found in this study are consistent with the literature and help push the understanding of metacognitive knowledge monitoring and executive functions forward: metacognitive knowledge monitoring is related to part of executive functions as it is found to be positively associated with the component executive function of updating, but not the other component executive functions in early adulthood.

A better understanding of the relationship of metacognition and executive functions is significant in both theoretical and practical perspectives. It helps explain the similarities found between these two theoretical constructs and brings new empirical evidence to further the understanding of the ambiguous and mixed results on their relationships. Practical teaching implications are also generated from the results. The relationship between updating and metacognitive monitoring suggests that more attention should be paid to updating skills in education. The improvement in updating skills is likely to facilitate metacognition and improve students’ learning outcomes. It suggests that a more promising result might be generated if updating skills is selected to be focus when attention cannot be paid to everything due to the limited time or resources. It also indicates the need for developing interventions to better practice students’ updating skills for higher learning achievements.

## 5. Limitations

We recognize that the small sample size and homogeneous sample (undergraduates at a large Midwestern university) limit the generalizability of our findings. Future studies should utilize larger and more diverse samples. Executive functions are known to develop at different rates. Samples with greater variability in age and executive functions might provide different results than those found with our limited sample of young adults. Large samples would also provide the opportunity to collect several measures of each construct in question and apply more advance statistical techniques, such as structural equation modeling, to allow for a more robust test of the relationships between constructs.

Another limitation of our study was the poor performance of participants on the incongruent trials of the Stroop task. The congruent trial accuracy was quite high and at the levels expected based on previous research. The incongruent trials were not at the levels to be expected and were indeed extremely low. We rechecked our program and did not find any errors in the program or data collection. A possible explanation is that participants were unclear about the instructions of the task. This is unfortunate as it did not allow for the appropriate analysis of the Stroop task (using the difference in reaction time between correct responses to congruent and incongruent trials). We do feel that our use of the difference between all congruent and incongruent trials, though not ideal, provided a measure of the ability to inhibit prepotent responses.

## 6. Conclusions

The results of the current study suggest that individual differences in knowledge monitoring accuracy in young adults are associated with individual differences in updating in working memory. It is our interpretation, metacognitive monitoring, as described by Nelson and Narens (1990), requires one to update the meta-level in the system by maintaining and updating contents in working memory related to the ongoing cognitive aspects of the task at hand. Future research should focus on more diverse samples and large samples sizes to replicate and extend our findings.

## Figures and Tables

**Table 1:** Means and standard deviations of JOK and JOC accuracy assessment results.

Variable	JOK Mean (SD)	JOC Mean (SD)
Hits	16.85 (6.48)	17.75 (6.19)
Correct Rejections	16.22 (4.93)	18.41 (5.57)
Misses	7.66 (4.51)	5.47 (522)
False Alarms	9.27 (3.93)	8.37 (4.02)
Accurate Monitoring (Hits + Correct Rejections)	33.07 (4.22)	36.16 (4.70)
Inaccurate Monitoring (Misses + False Alarms)	16.93 (4.22)	13.84 (4.70)
Gamma Coefficient	0.58 (0.24)	0.75 (0.28)

**Table 2:** Descriptive statistics of the results of the Stroop color–word task.

Variable	N	M	SD	Cronbach’s α
Congruent Acc	68	0.98	0.19	0.79
Congruent RT	68	726.33	128.70	0.82
Incongruent Acc	68	0.06	0.14	0.80
Incongruent RT	68	913.99	246.24	0.85
RT Difference	68	187.59	147.63	-

Note: RT Difference = the RT difference between incongruent and congruent trials.

**Table 3:** Descriptive statistics of the reaction time in the number–letter task.

Variable	N	M	SD	Cronbach’s α
RT—Consistent	65	798.73	237.76	0.92
RT—Inconsistent	65	1139.99	274.95	0.92
RT Difference	65	409.26	321.00	

**Table 4:** Correlations among outcome variables.

	MC–JOK Correlations	MC–JOC Correlations	JOK Gamma	JOC Gamma	ABCD Accuracy	ABCD RT	Number–Letter Shift Cost	Stroop RT Difference	Tower of Hanoi
MC–JOK Correlations	-	0.22	0.60 **	0.12	0.06	−0.25 *	−0.03	0.10	0.07
MC–JOC Correlations		-	0.25 **	0.53 **	0.04	0.29 *	0.20	−0.08	−0.07
JOK Gamma			-	0.23	0.24 *	−0.33 **	0.07	0.20	0.11
JOC Gamma				-	−0.01	−0.14	−0.06	0.02	−0.11
ABCD Accuracy					-	−0.65 **	−0.03	0.33 **	−0.03
ABCD RT						-	−0.09	−0.29 *	−0.06
Number–Letter Shift Cost							-	−0.06	0.04
Stroop RT Difference								-	−0.02
Tower of Hanoi									-

** *p* < 0.01 (2-tailed), * *p* < 0.05 level (2-tailed). Note: MC–JOK correlations = the mean correlation between multiple-choice accuracy and JOKs; MC–JOC correlations = the mean correlation between multiple-choice accuracy and JOCs.

**Table 5:** Regression coefficients with word accuracy JOK/JOC as the criterion.

**Word Accuracy/JOK**		**Standardized Coefficients**	**t**	**Sig.**	**Correlations**		
		**Beta**			**Zero-Order**	**Partial**	**Part**
	(Constant)		2.10	0.055			
	ABCD Acc	−0.16	−0.754	0.36	0.05	−0.13	−0.12
	ABCD RT	−0.33	−0.92	0.06	−0.21	−0.25	−0.25
	Number–Letter	−0.17	−0.87	0.22	−0.12	−0.17	−0.16
	TOH	0.11	0.69	0.42	0.12	0.11	0.11
	Stroop RT Difference	−0.02	−0.28	−0.16	0.03	−0.02	−0.02
**Word Accuracy/JOC**		**Standardized Coefficients**	**t**	**Sig.**	**Correlations**		
		**Beta**			**Zero-Order**	**Partial**	**Part**
	(Constant)		3.88	0.001			
	ABCD Acc	−0.36	−2.18	0.034	0.01	−0.29	−0.27
	ABCD RT	−0.53	−3.26	0.002	−0.33	−0.41	−0.40
	Letter–Number	0.12	0.98	0.330	0.17	0.13	0.12
	TOH	−0.10	−0.78	0.438	−0.04	−0.11	−0.10
	Stroop RT Difference	0.09	0.96	0.491	0.08	0.10	0.09

## Data Availability

Data are available on the Open Science Framework: https://osf.io/y5j6p/?view_only=4280ef15f61c4c67bd7fb9645ebf4f44, accessed on 20 November 2023.
